# Editorial: Genome edited organisms for agriculture—challenges and perspectives for development and regulation

**DOI:** 10.3389/fgeed.2023.1287973

**Published:** 2023-09-18

**Authors:** Michael Eckerstorfer, Sarah Zanon Agapito-Tenfen, Gijs A. Kleter

**Affiliations:** ^1^ Environment Agency Austria, Vienna, Austria; ^2^ Norwegian Research Institute (NORCE), Bergen, Norway; ^3^ Wageningen Food Safety Research, Wageningen University and Research, Wageningen, Netherlands

**Keywords:** genome editing, agriculture, risk assessment, traceability, regulation

With pleasure, we present this Research Topic of articles, which we believe will inform current and future discussions surrounding the regulation, traceability, and safety of genome-edited crops and derived food and feed products. A handful of genome edited-crops have already been commercialized in several nations across the globe and their number is likely to expand progressively in the coming few years. This prospect raises a number of questions, some of which are addressed in this Research Topic as follows:

• Are there any specific hazards inherent to this technology? To what extent are hazards similar to those of transgenic and conventional breeding? Besides the intended mutations, what does this mean for unintended effects, including those caused by so-called “off-target” mutations? Moreover, delivery methods still rely on classical genetic engineering methods such as biolistics and *Agrobacterium* transfection, but new methods are currently being developed which do not require the introduction of plasmid vectors.
o The work by Slaman et al. features a number of notable findings in this regard, indicating that the size of mutations introduced by CRISPR-Cas at target and off-target sites in tomato DNA was limited. Off-target mutations occurred at a much lower frequency, though, i.e. 5% of total reads at most, and more commonly below 1%. The authors transfected tomato protoplasts with plasmids encoding the CRISPR-Cas and sgRNAs targeting different *MYB* transcription factor genes. At 21 of the target sites, mutations were observed, particularly small insertions and deletions. A single-base pair (1-bp) insertion was the most favored single mutation. In addition, mutations at 194 predicted off-target sites occurred at a low frequency at sites with 1 or 2 mismatches to the sgRNA, but none at sites with 3 or 4 mismatches. These outcomes were also compared with the direct introduction of externally prepared ribonucleoproteins (RNPs) and sgRNA into protoplasts (without the use of plasmids).
• What would be the challenges for the enforcement of legislation for genome-edited crops?
o One of the challenges concerns the potential dissemination of imported, non-authorized genome-edited crops due to the different regulatory systems for genome-edited crops at the global level. Pascher et al. investigate a relevant case study on the spread of feral non-GM oilseed rape plants around transport routes (road, railroad), and importing and processing facilities in Austria during 2 years. Feral populations of varying sizes were indeed found at these places, indicative of seed spillage. Large populations were particularly found where oilseed rape is handled, as were potentially cross-breeding wild relatives in their vicinity. The genetic variety was the greatest at port and mill sites, whilst variety was greater within than between populations. This shows that if genome-edited oilseed is accidentally spilled, it could establish within such feral groups and the environmental risks would therefore have to be considered. In addition, the authors point to the lack of information on GM crop varieties from developers and exporting countries, which hampers a comprehensive monitoring strategy, and to the lack of appropriate detection methods for some genome-edited varieties.
o Another challenge in some legislative frameworks is the determination of the regulatory status of so-called null-segregants or negative segregants. For genome editing with site-directed nucleases such as CRISPR-Cas, transgenes encoding the CRISPR tools are often inserted in the plant genome and subsequently removed, either through segregation or excision using, e.g., the Cre Lox system. Using the definition of living modified organisms from the Cartagena Protocol, the international treaty on GMOs, under the Convention on Biological Diversity as a starting point, Heinemann et al. explore whether null segregants should be considered genetically modified organisms and therefore should be regulated. Their analysis shows that null segregants are covered by the GMO laws in many countries which have accommodated this international definition since they are organisms with new combinations of genetic material created by techniques of modern biotechnology. They also contend that even if null-segregants are to be deregulated through exclusion or exemption from the requirements of GMO legislation, null segregants are hazards if in some environments unintended changes or unintended outcomes of intended changes lead to adverse effects. And even when there should be checks to ensure that indeed no transgenic DNA is still present within the plant, null segregants may still carry unintended legacy insertions of DNA contaminating the commercial formulations in which genome editing reagents are supplied or have undetected legacy insertions and deletions outside the insertion region. Finally, the authors discuss the effects of scale with regard to the risks of the widespread use of genome editing and indicate that the likelihood of occurrence of harmful incidents related to unintended and unforeseen effects of genome editing may increase when null-segregants are used more broadly.
o The distinction between the different types of modification that can be brought about by genome editing is an important issue in current regulatory discussions. Instead of a categorical exemption or full regulation, Voigt proposes a tiered approach toward the regulation of genome-edited crops in the EU based on the nature of the modifications. The first tier applies to plants with non-transgenic modifications that are identical to what can be achieved through conventional and natural means, based on a pre-set list of eligible edit types. These only require a notification and proof that no other modifications are still present, but no further data requirements. For the second tier, there is still an absence of transgenes, such as in plants with certain multiple DNA edits, cisgenic or intragenic DNA insertions, or novel phenotypes. The authors propose a decision procedure based on the outcomes of a risk-screening of compositional data and the familiarity with the introduced trait. It is then decided if an authorization and risk assessment for GMOs will be necessary on a case-by-case basis. The third tier consists of plants that contain transgenes and for which the full authorization requirements according to the GMO legislation would be required.

We believe that these contributions will broaden the scope of current discussions and inform the development of policies toward the safe and responsible use of genome editing in agriculture.

